# A Scientific Smuggler

**Published:** 1882-05

**Authors:** 


### A SCIENTIFIC SMUGGLER.

The Berlin *Montagsblatt* tells this story : In 1805 Humboldt and Guy-Lussac were in Paris, engaged in experiments on the compression of air. The two scientists found themselves in need of a large number of glass tubes. These were exceedingly dear in France at the time, and the rate of import was something alarming. Humboldt sent to Germany for the needed articles, and gave directions that the manufacturer should seal up the tubes at both ends, and put a label upon each tube with the words *Deutsche Luft* ("German air"). The air of Germany was an article upon which there was no duty, and the tubes were passed by the custom officers without any demand, and arrived free of duty in the hands of the two experimenters.

